# Electrophysiological characterization of the modified hERG_T_ potassium channel used to obtain the first cryo‐EM hERG structure

**DOI:** 10.14814/phy2.14568

**Published:** 2020-10-22

**Authors:** Yihong Zhang, Christopher E. Dempsey, Jules C. Hancox

**Affiliations:** ^1^ School of Physiology and Pharmacology and Neuroscience Biomedical Sciences Building The University of Bristol University Walk Bristol UK; ^2^ School of Biochemistry Biomedical Sciences Building The University of Bristol University Walk Bristol UK

**Keywords:** hERG, I_Kr_, KCNH2, Kv11.1, Long QT Syndrome, LQT2, potassium channel, rapid delayed rectifier

## Abstract

The voltage‐gated hERG (*human‐Ether‐à‐go‐go Related Gene*) K^+^ channel plays a fundamental role in cardiac action potential repolarization. Loss‐of‐function mutations or pharmacological inhibition of hERG leads to long QT syndrome, whilst gain‐of‐function mutations lead to short QT syndrome. A recent open channel cryo‐EM structure of hERG represents a significant advance in the ability to interrogate hERG channel structure‐function. In order to suppress protein aggregation, a truncated channel construct of hERG (hERG_T_) was used to obtain this structure. In hERG_T_ cytoplasmic domain residues 141 to 350 and 871 to 1,005 were removed from the full‐length channel protein. There are limited data on the electrophysiological properties of hERG_T_ channels. Therefore, this study was undertaken to determine how hERG_T_ influences channel function at physiological temperature. Whole‐cell measurements of hERG current (I_hERG_) were made at 37°C from HEK 293 cells expressing wild‐type (WT) or hERG_T_ channels. With a standard +20 mV activating command protocol, neither end‐pulse nor tail I_hERG_ density significantly differed between WT and hERG_T_. However, the I_hERG_ deactivation rate was significantly slower for hERG_T_. Half‐maximal activation voltage (V_0.5_) was positively shifted for hERG_T_ by ~+8 mV (*p* < .05 versus WT), without significant change to the activation relation slope factor. Neither the voltage dependence of inactivation, nor time course of development of inactivation significantly differed between WT and hERG_T_, but recovery of I_hERG_ from inactivation was accelerated for hERG_T_ (*p* < .05 versus WT). Steady‐state “window” current was positively shifted for hERG_T_ with a modest increase in the window current peak. Under action potential (AP) voltage clamp, hERG_T_ I_hERG_ showed modestly increased current throughout the AP plateau phase with a significant increase in current integral during the AP. The observed consequences for hERG_T_ I_hERG_ of deletion of the two cytoplasmic regions may reflect changes to electrostatic interactions influencing the voltage sensor domain.

## Graphical abstract

The hERG_T_truncation mutant was made to derive the cryo‐EM structure that is employed to study hERG K^+^channel structure function. This paper shows that the hERG_T_ mutant exhibits modest differences in hERG current kinetics from the wild‐type channel. Voltage‐dependent activation and “window current” were positively voltage‐shifted, deactivation was slowed and recovery from inactivation was faster for hERG_T_ compared to the wild‐type channel.

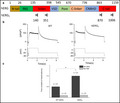

## 1 INTRODUCTION

Cardiac action potential repolarization involves the coordinated activity of several key potassium (K^+^) ion channels (Tamargo, Caballero, Gomez, Valenzuela, & Delpon, 2004). The rapid delayed rectifier K^+^ current, I_Kr_, plays an important role in repolarization from ventricular action potential (AP) plateau voltages, with current progressively increasing during the plateau before declining during terminal repolarization (Hancox, Levi, & Witchel, 1998; Mitcheson & Hancox, 1999; Rocchetti, Besana, Gurrola, Possani, & Zaza, 2001; Sanguinetti & Tristani‐Firouzi, 2006). This final repolarization phase is mediated by the distinct, inwardly rectifying K^+^ current, I_K1_ (Mitcheson & Hancox, 1999; Shimoni, Clark, & Giles, 1992; Zaza, Rochetti, Brioschi, Cantadori, & Ferroni, 1998). In addition to contributing to repolarization during APs, I_Kr_ can regulate diastolic depolarization of cells in pacemaker regions of the cardiac conduction system, through continued deactivation of I_Kr_ following AP repolarization (Mitcheson & Hancox, 1999; Ono & Ito, 1995). I_Kr_ is carried by channels encoded by *human‐Ether‐à‐go‐go Related Gene* (*hERG*, alternative nomenclature *KCNH2*) (Sanguinetti, Jiang, Curran, & Keating, 1995; Trudeau, Warmke, Ganetzky, & Robertson, 1995). hERG channel current (I_hERG_), like I_Kr_, is characterized by rapid voltage‐dependent inactivation, which plays a critical role in shaping the channel's contribution to ventricular AP repolarization (Hancox, McPate, El Harchi, & Zhang, 2008; Sanguinetti & Tristani‐Firouzi, 2006; Vandenberg, Walker, & Campbell, 2001). The channel's relatively fast inactivation/recovery from inactivation kinetics together with slower deactivation kinetics also means that hERG/I_Kr_ channels can generate outward transient currents that oppose premature stimuli late in repolarization/early in diastole (Lu et al., 2001; Vandenberg et al., 2001).

**Figure 1:**
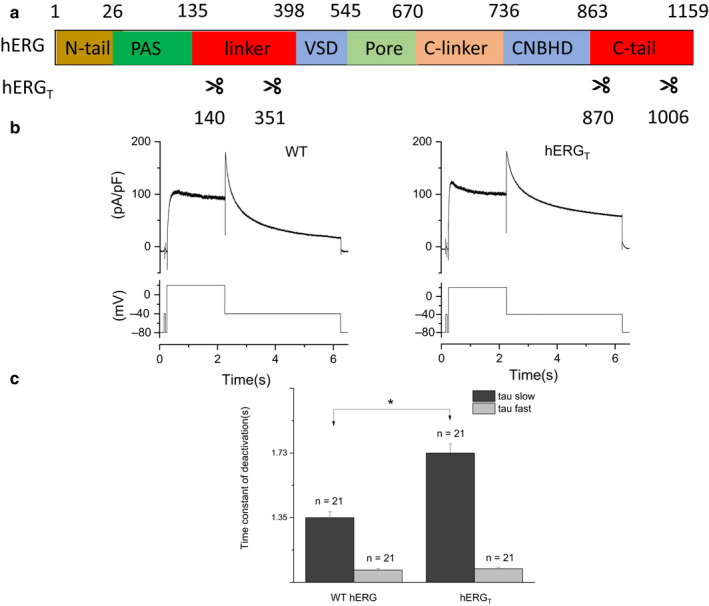
hERG_T_ structure, current profile, and deactivation. (a) Schematic diagram showing truncated hERG_T_ compared to WT hERG domains (indicated by use of different colors). In hERG_T_, amino acid residues between 140–351 and 870–1006 were removed (regions encoding residues 141–350 and 871–1005 were removed). (b) Example current (I_hERG_) traces for WT hERG (left panel) and hERG_T_ (right panel) with corresponding voltage protocol shown in the lower panels. (c) Bar chart shows comparison of fast and slow deactivation time constants between WT and hERG_T_ (*n* = 21 for each of WT and hERG_T_, * denotes statistical significance of *p* < .05; unpaired *t* test)

Given the important physiological roles of hERG/I_Kr_ channels, it is unsurprising that *hERG* mutations have adverse consequences. Loss‐of‐function *hERG* mutations underpin the LQT2 form of congenital long QT syndrome (LQTS), whilst gain‐of‐function mutations underlie the SQT1 form of congenital short QT syndrome (SQTS); both conditions predispose to malignant arrhythmias and sudden death (Hancox, Whittaker, Du, Stuart, & Zhang, 2018; Sanguinetti & Tristani‐Firouzi, 2006). hERG channels also have a remarkable sensitivity to pharmacological inhibition by diverse pharmaceuticals linked to the drug‐induced form of acquired LQTS (Hancox et al., 2008; Sanguinetti & Tristani‐Firouzi, 2006). Due to this, novel pharmaceuticals must be tested for activity against the hERG channel (Gintant, 2008; Hancox et al., 2008). Current understanding of hERG channel structure‐function has derived from a combination of functional mutagenesis studies and in silico modeling. For most of the period since the first electrophysiological studies of hERG in 1995 (Sanguinetti et al., 1995; Trudeau et al., 1995), in silico reconstructions of hERG structure have relied on homology modeling; in 2017 this changed with the publication of the first cryo electron microscopy (cryo‐EM)‐derived hERG structure (Wang & MacKinnon, 2017). This structure, of an open channel with voltage sensors captured in a depolarized conformation, provides unprecedented opportunities to better understand hERG channel gating and pharmacology (Butler, Helliwell, Zhang, Hancox, & Dempsey, 2020; Robertson & Morais‐Cabral, 2019). It is important to note, however, that because the full‐length wild‐type (WT) hERG protein tends to aggregate during purification (Su, Brown, Wang, & MacKinnon, 2016; Wang & MacKinnon, 2017), the wild‐type hERG construct used to obtain the cryo‐EM structure contained deletions of two segments of the channel (see Figure 1a; residues 141–350 and 871–1005), each predicted to be disordered (Wang & MacKinnon, 2017). I_hERG_ kinetics data for the truncated construct, hERG_T_, are limited to date, showing a modest (+5 mV) shift in voltage‐dependent activation, whilst the channel retained sensitivity to inhibition by the high affinity inhibitors dofetilide and astemizole (Wang & MacKinnon, 2017). A question arises, therefore, as to the extent to which the kinetics of I_hERG_ carried by hERG_T_ resemble or differ from those of the intact WT channel. Consequently, this study was undertaken to compare I_hERG_ carried by intact hERG with that carried by hERG_T_.

## 2 METHODS

### 2.1 Mutagenesis

The hERG_T_ 814 amino acid truncation construct in which two segments of the complete 1,159 amino acid protein (141–350 and 871–1005) were deleted (Wang & MacKinnon, 2017), was generated by Mutagenex Inc (Suwanee, GA 30,024, USA) from a WT hERG construct template in a vector of modified pcDNA3 used in our laboratory. Competent DH5*α Escherichia coli* (Invitrogen, Paisley, UK) were transformed using standard procedures, DNA was purified using a Endotoxin‐free plasmid DNA purification kit (Neumann‐Neander‐Str., Germany, Macherey‐Nagel), and the mutation was confirmed by sequencing of the entire open reading frame (Eurofins MWG Operon, Ebersberg, Germany).

### 2.2 Cell culture and transfection

Human embryonic kidney (HEK 293) cells (European Collection of Cell Cultures, Porton Down, UK) were used to study the effects of the WT and hERG_T_ on I_hERG_ kinetics and profile under action potential (AP) voltage clamp. These cells were maintained at 37°C, 5% CO_2_ in Dulbecco's minimum essential medium with Glutamax‐1 (DMEM; Gibco, Paisley, UK). This was supplemented with 10% fetal bovine serum. Cells were transiently transfected with 1μg of cDNA plasmids encoding WT or hERG_T_ using Lipofectamine 2000 (Invitrogen, Paisley, UK) according to the manufacturer's instructions. Expression plasmid encoding CD8 (0.15μg) was also added (in pIRES, donated by Dr I Baró, University of Nantes, France) to be used as a successful marker of transfection. Successfully transfected cells (positive to CD8) were identified using Dynabeads® (Invitrogen, Paisley, UK). This approach, which utilizes polystyrene microspheres coated with CD8 antibody that adhere to CD8^+^ cells, has long been proposed to be valuable for visual identification of transfected cells for electrophysiology experiments (Jurman, Boland, Liu, & Yellen, 1994). Electrophysiological recording experiments were performed 12–48 hr after transfection (a range within that of prior studies from our laboratory; Butler, Zhang, Stuart, Dempsey, & Hancox, 2019; Melgari et al., 2015; Zhang et al., 2016).

### 2.3 Solutions for electrophysiological recordings

Once the coverslip containing cells was put in the recording chamber, cells were superfused with normal Tyrode's containing (in mM): 140 NaCl, 4 KCl, 2.5 CaCl_2_, 1 MgCl_2_, 10 Glucose, and 5 HEPES (titrated to pH of 7.45 with NaOH) (Butler et al., 2019; Melgari et al., 2015; Zhang et al., 2016). The pipette dialysis solution for hERG current (I_hERG_) measurement contained (in mM): 130 KCl, 1 MgCl_2_, 5 EGTA, 5 MgATP, and 10 HEPES (titrated to a pH of 7.2 with KOH) (Butler et al., 2019; Melgari et al., 2015; Zhang et al., 2016).

### 2.4 Experimental protocols

Whole‐cell conventional and human AP voltage clamp (“AP clamp”) recordings of I_hERG_ were made at 37 ± 1°C by using an Axopatch 200B amplifier (Axon Instruments, Foster City, CA, USA). Patch pipettes were fire polished to 2.5–4 MΩ. Between 70% and 80% of the electrode series resistance could be compensated. Data were recorded via a Digidata 1440A interface (Molecular Devices, Sunnyvale, CA, USA). Data digitization rates were 10–25 kHz during all protocols and an appropriate bandwidth of 2–10kHz was set on the amplifier. Currents elicited under “AP clamp” were corrected online for P/N leak subtraction using an interspersed P/4 protocol (Butler, Zhang, Stuart, Dempsey, & Hancox, 2018; McPate et al., 2009; Melgari et al., 2015). The specific voltage protocols used experimentally are detailed within the relevant figures and associated Results.

Half‐maximal activation (V_0.5_) voltage values were obtained by normalizing I_hERG_ tail values (I) at −40 mV following differing voltage commands to the maximal I_hERG_ tail value observed during the voltage protocol (Imax). The resulting values were plotted against corresponding command voltage (Vm), and fitted by a Boltzmann equation of the form:

(1) $$I/I\max=1/\left(1+\exp\left(\left(V_{0.5}‐\text{Vm}\right)/k\right)\right)$$

Half‐maximal inactivation voltage (V_0.5_) was obtained from normalized plots of voltage‐dependent availability, using the following equation:

(2) $$\text{Inactivation parameter}=1‐\left(1/\left(1+\exp\left[\left(V_{0.5}‐\text{Vm}\right)/k\right]\right)\;\right)$$

Where the inactivation parameter occurs within the range 0 – 1, Vm represents the repolarization voltage used to influence I_hERG_ availability, V_0.5_ is the half‐maximal inactivation voltage and *k* is the slope factor describing the I_hERG_ inactivation relation.

Continuous plots of voltage‐dependent activation and inactivation relations were obtained from half‐maximal activation/inactivation voltage (V_0.5_) and slope factor (*k*) values derived from experimental data, by calculation of activation and inactivation parameter values at 2 mV intervals between −150 and +100 mV, using equations 1 and 2 (Colenso, Sessions, Zhang, Hancox, & Dempsey, 2013; Zhang, Colenso, Sessions, Dempsey, & Hancox, 2011).

Action potential (AP) clamp experiments were conducted, as described previously, using a human epicardial ventricular AP waveform generated by the ten Tusscher–Noble–Noble–Panfilov ventricular tissue model (ten McPate et al., 2009; Tusscher, Noble, Noble, & Panfilov, 2004).

### 2.5 Data presentation and statistical analysis

Data were analyzed using Clampfit 10.2 (Axon Instruments), Excel 2016 (Microsoft, Redmond, WA), Origin 2018b (OriginLab Corporation, Northampton, MA, USA), and Prism 8 (Graphpad Inc, La Jolla, CA, USA) software. Total charge carried by WT and hERG_T_ channels during AP commands was determined by integrating currents using Origin 2018b (Butler et al., 2018). Statistical comparisons were made using the Student's *t* test, or two way analysis of variance (ANOVA) followed by Bonferroni *post hoc* test, as appropriate. *P* values less than 0.05 were taken as being statistically significant.

## 3 RESULTS

### 3.1 I_hERG_ carried by hERG_T_ channels during a standard voltage “step” protocol

Figure 1a illustrates the truncated hERG_T_ construct compared with WT hERG. The first deleted region encoding residues 141–350 (cut between 140 and 351), removed most of the N‐linker region that connects the N terminal Per‐ARNT‐Sim (PAS) domain with S1 of the voltage sensor domain (VSD); the second deleted region encoding residues 871–1005 (cut between 870 to 1,006), eliminated much of the long cytoplasmic C terminal tail that follows the cytoplasmic cyclic nucleotide binding homology domain (CNBHD). In initial experiments, the profile of I_hERG_ was compared between WT and hERG_T_ channels using a standard I_hERG_ protocol comprised of a 2 s depolarization from −80 to +20 mV, followed by repolarization to −40 mV. A brief (50 ms) prepulse from −80 to −40 mV preceded the +20 mV command, to provide a reference value for I_hERG_ tail amplitude measurement (Melgari et al., 2015; Zhang et al., 2016) WT I_hERG_ (Figure 1b, left panel) exhibited well‐established characteristics: current development during the applied depolarization with a resurgent I_hERG_ “tail” elicited during a repolarizing step to −40 mV. I_hERG_ carried by hERG_T_ (Figure 1b, right panel) exhibited a similar overall profile. The I_hERG_ tail and end‐pulse current density were respectively 151.40 ± 22.28 pA/pF and 83.43 ± 13.93 pA/pF for hERG_T_ (*n* = 21); for WT hERG the comparable values were 169.48 ± 25.10 pA/pF and 81.92 ± 12.65 pA/pF, respectively (*n* = 21). There was no significant difference in either value between WT and hERG_T_ (*p* = .59 and 0.94 for tail and end‐pulse current density respectively, Student's *t* test). Tail current deactivation was fitted with a biexponential function to derive fast and slow (τ_f_ and τs) time constant values (Zhou et al., 1998) (Figure 1c). The mean τ_s_ value (describing the slow component of deactivation) was significantly larger for I_hERG_ carried by hERG_T_ than WT channels (1.73 ± 0.14 s for hERG_T_ and 1.35 ± 0.09 s for WT, *p* < .05; *n* = 21 for each, Student's *t* test). τ_f_ and the relative proportion of fast/slow deactivation showed no significant change compared to WT (*p = *.63 and 0.59 respectively, Student's *t* test). Thus, hERG_T_ slowed I_hERG_ deactivation by increasing τs.

### 3.2 WT and hERG_T_ current–voltage (I–V) relationships compared

**Figure 2:**
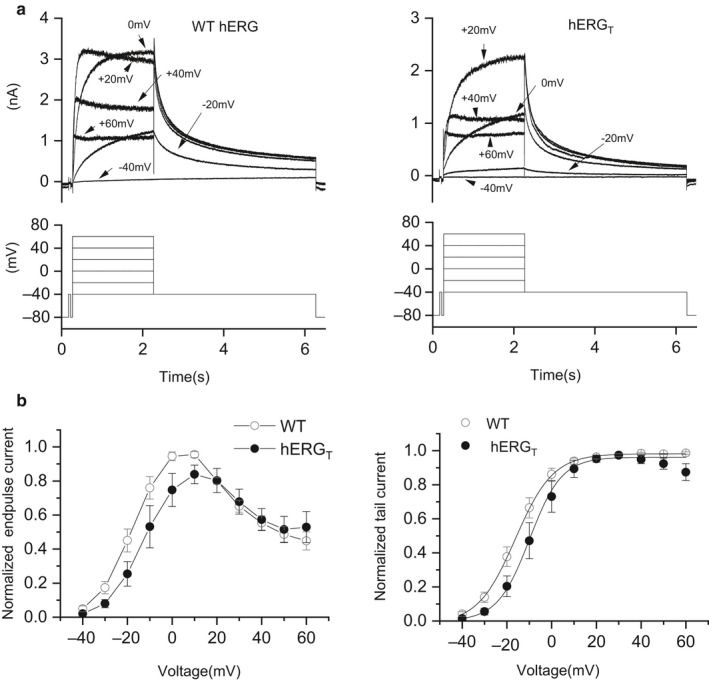
Current–voltage (I–V) relationship for WT and hERG_T_ I_hERG. _(a) Upper panels show representative I_hERG_ families of currents for WT hERG (left panel) and hERG_T_ (right panel). Test pulses were applied at 10 mV increments between −40 mV and +60 mV, with only selected traces being shown for clarity of display. Corresponding test potentials of the voltage protocol are indicated in the lower panel (start‐to‐start interval of 12 s between successive voltage steps). (b) Mean I–V relations for end‐pulse currents (left panel) and mean normalized tail currents I–V relations (right panel); for each of WT (*n* = 9) and hERG_T_ (*n* = 7), data were normalized to the maximal currents recorded during the protocol. Tail current data were fitted with equation 1 to give the V_0.5_ and *k* values provided in the Results text.

The voltage dependence of WT and hERG_T_ I_hERG_ was compared using 2 s depolarizing voltage steps from a holding potential of −80 mV to potentials between −40 and +60 mV (Butler et al., 2019; Zhang et al., 2011). Representative traces at selected potentials are shown in Figure 2a for WT (upper left panel) and hERG_T_ (upper right panel) I_hERG_, with the corresponding voltage protocol underneath. The normalized mean I–V data are shown in Figure 2b. For WT I_hERG,_ the end‐pulse current increased progressively with depolarization up to ~ 0mV, declining after 10 mV, giving rise to a well‐established bell shaped current–voltage (I–V) relation (Figure 2b, left panel). For hERG_T_, the I–V relation showed a similar profile, but peaked at +10 mV, with an apparent modest rightward shift of the end‐pulse I–V relation compared to WT hERG. 2‐way ANOVA analysis did not reveal overall significant differences between WT and hERG_T_ in the normalized end‐pulse I–V relations (*p* = .11). However, such analysis did show an overall significant difference between WT and hERG_T_ in the normalized tail I–V relations (*p* < .05), although the conservative Bonferroni post‐test did not identify significance at particular potentials in the tested range.

Scrutiny of the representative traces for WT and hERG_T_ I_hERG_ in Figure 2a shows that for test voltages up to ~0 mV, hERG_T_ I_hERG_ was activated to smaller extent than was that for WT hERG. Normalized I–V relations for I_hERG_ tails were used to quantify voltage‐dependent activation, with fits to equation 1 used to derive half‐maximal activation voltage (V_0.5_) and slope (*k*) values (right panel of Figure 2b). The V_0.5_ and *k* values for WT I_hERG_ activation derived from the fits were −15.96 ± 1.89mV and 7.52 ± 0.55mV (*n* = 9). For hERG_T_ the comparable values were: −8.29 ± 3.10mV and 5.47 ± 1.01mV, respectively (*n* = 7; *p* < .05 for V_0.5_ and *p* = .11 for *k* versus WT, Student's *t* test). Thus, the V_0.5_ describing the voltage dependence of activation of I_hERG_ for hERG_T_ was significantly shifted by ~+8mV compared to the WT channel (*cf* ~+5 mV reported by Wang & MacKinnon, 2017). This rightward shift in voltage‐dependent activation of hERG_T_ was associated with a slower rise‐time of currents during the activating command at some voltages. Thus, exponential fitting of the current activated by the test pulse to −10 mV yielded rise‐time τ values of 930.85 ± 133.78 ms for hERG_T_ and 554.26 ± 75.84 ms for WT hERG (*p* < .05). During the command to 0 mV, the rise‐time τ was 577.61 ± 113.09 ms for hERG_T_ and 296.28 ± 56.92 ms for WT (*p* < .05). At + 20 mV, however, there was no significant difference between hERG_T_ (188.39 ± 64.30 ms) and WT hERG (78.27 ± 6.77 ms; *p* = .07).

### 3.3 Comparison of inactivation characteristics between WT and hERG_T_ I_hERG_

**Figure 3:**
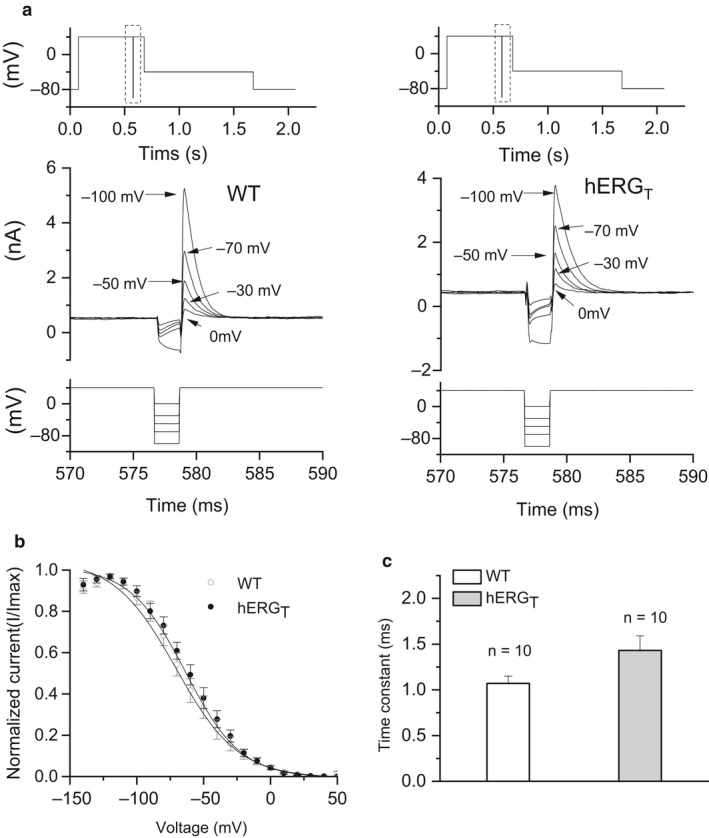
Voltage and time‐dependence of inactivation for WT and hERG_T_ I_hERG_. (a) Upper panels show the whole protocol used to interrogate inactivation, with the highlighted area shown enlarged in the main panels. Main panels show representative traces of WT (Left panel) and hERG_T_ (right panel) I_hERG_, focusing on the current profile during the second and start of the third steps of the protocol, only selected traces shown for clarity of display. Relevant portion of voltage protocol shown below currents. (b) I_hERG_ availability plots for WT (*n* = 10) and hERG_T_ (*n* = 10). Data were fitted with equation 2 to give the V_0.5_ and *k* values presented in the Results text. (c) Bar chart showing the time constant for development of inactivation of WT (*n* = 10) and hERG_T_ I_hERG_ (*n* = 10) obtained by single exponential fitting of the currents elicited following the repolarizing step to −120 mV.

We proceeded to determine the voltage dependence of I_hERG_ inactivation (availability) by using the protocol shown in the upper panel of Figure 3a, in the protocol (Zhang et al., 2011; Butler *et al*., 2019). An initial depolarizing command to +40 mV was used to activate and then inactivate I_hERG_; following this, a 2 ms brief repolarizing command was applied to a range of potentials (in 10 mV increments down to −140 mV) to relieve inactivation to varying extents. This was followed by a third step to +40 mV. The magnitude of peak current elicited by the third step reflected the extent of availability induced by the second step (Butler et al., 2019; Zhang et al., 2011). Figure 3a shows representative traces from WT (middle left panel) and hERG_T_ I_hERG_ (middle right panel) respectively. To correct for possible deactivation during this protocol as in previous work from our laboratory, a method described by Zou *et al* was used (McPate, Duncan, Milnes, Witchel, & Hancox, 2005; Zhang et al., 2011; Zou, Xu, & Sanguinetti, 1998). Peak current amplitudes during the third pulse were obtained by single exponential fitting of the currents and extrapolation to the start of the third step (McPate et al., 2005; Zhang et al., 2011; Zou et al., 1998). Peak currents during the third step were then normalized to maximal current and mean data were plotted as shown in Figure 3b, Boltzmann fitting this relation, using equation 2, yielded an inactivation V_0.5_ value of −66.33 ± 5.38 mV (*k* = 18.27 ± 1.69; *n* = 10) for WT and −59.99 ± 3.92 mV for hERG_T_ I_hERG_ (*k* = 19.96 ± 2.26, *n* = 10, *p* > .05 for V_0.5_ (*p* = .35) and for *k* (*p* = .56) compared with WT).

The time course of development of inactivation was quantified by mono‐exponential fitting of the decline of I_hERG_ transients following repolarization steps to −120 mV in this protocol. The inactivation τ‐values obtained from this are shown in Figure 3c; these were 1.07 ± 0.08 ms (*n* = 10) and 1.43 ± 0.16 ms for WT and hERG_T_ I_hERG_ respectively (*n* = 10). Although there was a trend towards a slowed inactivation time course for hERG_T_ I_hERG_, this did not attain statistical significance (*p* = .07, Student *t* test).

**Figure 4:**
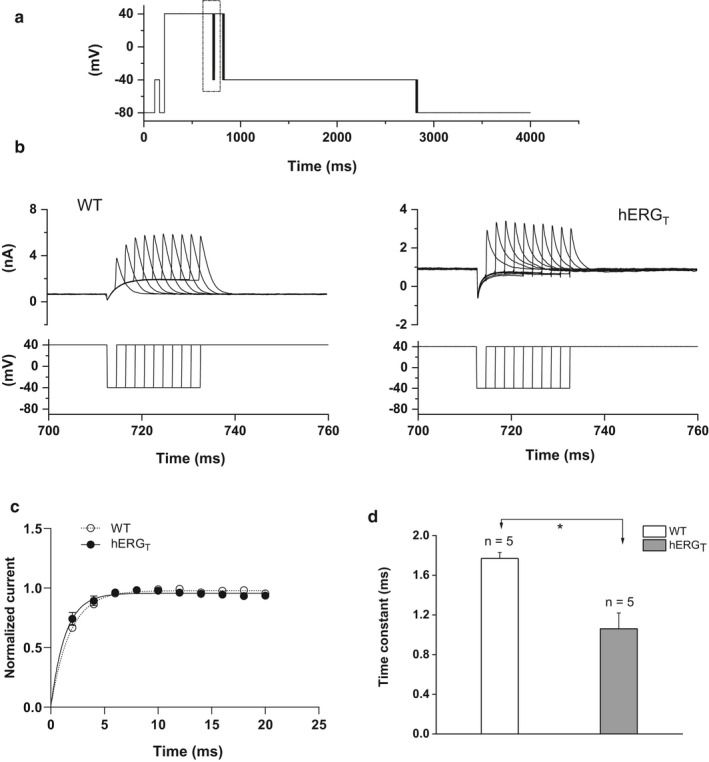
Time course of recovery from inactivation for WT and hERG_T. _(a) Whole protocol used to assess the time course of recovery of I_hERG_ from inactivation, with highlighted area shown in the lower panel of panel 4B. (b) Representative traces of WT and hERG_T_ I_hERG_ with corresponding portion of the voltage protocol shown below the current traces. (c) Mean normalized current plot with time. The dashed line denotes mono‐exponential fit to WT data (open circles, *n* = 5). The solid line denotes mono‐exponential fit to hERG_T_ (filled circles, *n* = 5). (d) Bar chart showing the comparison of recovery time constants for WT (*n* = 5) and hERG_T_ I_hERG_ (*n* = 5, * denotes statistical significance of *p* < .05; Student's *t* test)

The time course of recovery of I_hERG_ from inactivation was assessed using a protocol (Figure 4) in which a depolarization step to +40 mV was first applied for 500 ms to activate and inactivate I_hERG_; this was followed by a repolarisation step to −40 mV (a voltage close to which peak I_hERG_ occurs during physiological repolarization (McPate et al., 2009)) for increasing periods of time (between 2 and 20 ms) to release inactivation. A second depolarization step to +40 mV was then applied for 100 ms and the I_hERG_ measured, (Figure 4a). From this, the rate of recovery of I_hERG_ from inactivation was quantified by mono‐exponentially fitting the transient peak currents following different duration steps to  −40 mV (McPate et al., 2009; Zhang et al., 2011). Representative traces for WT and hERG_T_ I_hERG_ during this protocol are shown in Figure 4b with the lower panel showing the corresponding voltage protocol. The initial two hERG_T_ records with this protocol (upper right panel of Figure 4b) attained a greater proportion of maximal current amplitude than was the case for WT hERG (upper left panel of Figure 4b), suggestive of faster recovery from inactivation. Peak outward transient currents were normalized to the maximal current observed during the protocol and the resulting mean data were plotted against the duration of the repolarization step. These relations were fitted with a mono‐exponential function, giving τ values of 1.77 ± 0.06 ms for WT (*n* = 5) and 1.06 ± 0.16 ms for hERG_T_ (*n* = 5; *p* < .05, Student's *t* test). Thus, hERG_T_ I_hERG_ exhibited significantly accelerated recovery of inactivation.

**Figure 5:**
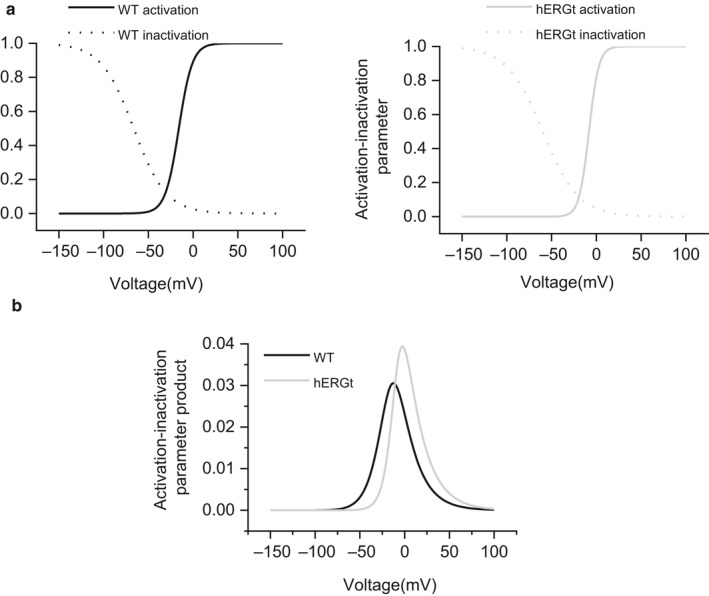
WT and hERG_T_ window current. (a) Superimposed activation (solid line) and inactivation (availability, dashed line) relations for WT (black, left panel) and hERG_T_ (gray, right panel) I_hERG_ respectively. Activation and inactivation parameters were calculated at 2‐mV intervals, using the V_0.5_ and *k* values obtained from fitting the experimental data. (b) “Window current” (the product of activation‐inactivation parameters) plotted against membrane potential to show the steady‐state WT (in black) and hERG_T_ (in gray) I_hERG_ window.

The V_0.5_ and *k* values obtained from the activation and inactivation fits to experimental data were used to calculate activation and inactivation parameters for I_hERG_ carried by WT and hERG_T_ over a wide range of voltages, as plotted in Figure 5a, left and right panel, respectively. The product of activation and inactivation parameters at each membrane potential was calculated and plotted as shown in Figure 5b, in order to obtain “window” current for WT hERG and hERG_T_ (Butler et al., 2019; Zhang et al., 2011). The I_hERG_ window was slightly rightward shifted and exhibited a modest increase in the peak of the window for hERG_T_.

### 3.4 I_hERG _profile during ventricular AP clamp compared between WT and hERG_T_

**Figure 6:**
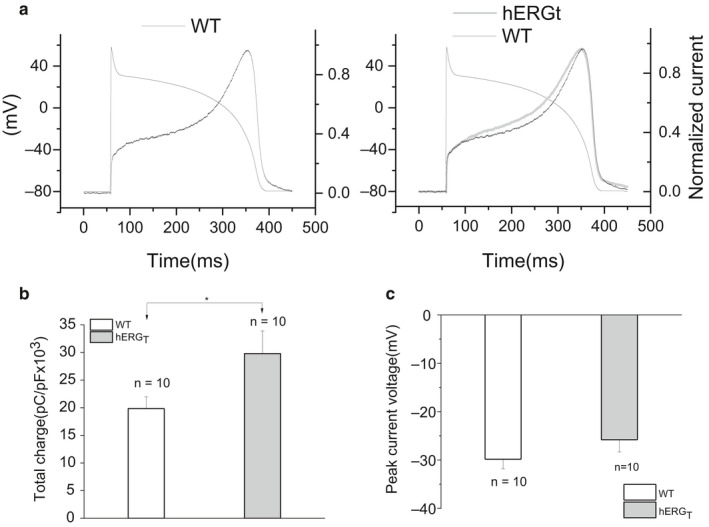
I_hERG_ during AP clamp for WT and hERG_T._ (a) Ventricular AP command waveform overlying mean normalized value of I_hERG_ for WT (left panel in black, *n* = 10) and hERG_T_ (right panel in gray, *n* = 10, with corresponding WT current superimposed in black). (b) Comparison of the total charge carried by each channel (*n* = 10 for each) during each action potential, calculated by integrating the current traces, normalized to cell capacitance, * denotes statistical significance of *p* < .05; Student's *t* test. (c) Comparison of the voltage at which the peak currents occurred during AP repolarization for each of WT and hERG_T_ I_hERG_

The AP clamp technique enables membrane potential “history” to be taken into account during dynamic activation of an ionic current of interest (Hancox et al., 1998; Noble, Varghese, Kohl, & Noble, 1998; Zhou et al., 1998). It therefore allows currents to be measured with their normal physiological time course and voltage dependence. Figure 6a shows mean normalized I_hERG_ traces for WT (left panel) and hERG_T_ (gray trace in right panel with WT current superimposed in black), elicited by the ventricular AP command shown. WT I_hERG_ was initially small, then increased progressively through the AP plateau phase up to a peak before declining during terminal AP repolarisation; the maximal current for WT hERG occurred at −29.84 ± 1.98mV (*n* = 10) (Hancox et al., 1998; McPate et al., 2005, 2009). The mean normalized I_hERG_ for hERG_T_ appeared to be bigger throughout the AP plateau phase, peaking slightly earlier during the AP. We measured the current integral (area under curve, normalized to cell capacitance) for each channel. Figure 6b compares the total charge carried by WT and hERG_T_ under AP clamp, the current integral for hERG_T_ was significantly larger than that for WT hERG (*p* < .05, Student's *t* test, *n* = 10 for each). While plots of the mean voltage at which maximal current was recorded during repolarization (Figure 6c) showed a modest shift of ~+4 mV (to −25.81 ± 2.51 mV; *n* = 10) for hERG_T_ compared to the WT channel, this difference did not attain statistical significance (*p* = .25, Student's *t* test).

## 4 DISCUSSION

To our knowledge, the only prior information on the electrophysiological properties of the hERG_T_ deletion construct comes from the original cryo‐EM study (Wang & MacKinnon, 2017), reporting a +5mV shift in activation V_0.5_ at ambient temperature. Here, we observed a ~+8mV activation V_0.5_ shift at 37°C, together with a rightward shift in window current, slowed deactivation, slightly accelerated recovery from inactivation, and increased current integral during an applied AP command. Several aspects of these findings are worthy of discussion.

### 4.1 Relating observed changes in kinetics to channel structure?

Similar to other voltage‐gated K^+^ channels, functional hERG channels are comprised of a tetramer of subunits containing six transmembrane segments, with a voltage sensor domain (VSD) comprised of S1–S4 segments. In contrast to many other Kv channels, however, the cryo‐EM structure of hERG shows a lack of domain‐swapping and the VSD is in close apposition to the pore domain of the same subunit (Butler et al., 2020; Robertson & Morais‐Cabral, 2019; Wang & MacKinnon, 2017). Voltage‐dependent inactivation occurs at the outer mouth of the channel, with comparison of hERG_T_ and hERG_TS_‐S631A (impaired inactivation) mutant structures showing subtle differences in the position of side chains at the outer portion of the selectivity filter (Robertson & Morais‐Cabral, 2019; Wang & MacKinnon, 2017).

Deletion of the N terminal hERG‐specific domain between residues 138–373 has previously been reported to produce a negative shift in voltage‐dependent activation of I_hERG_, (Viloria, Barros, Giraldez, Gomez‐Varela, & de la Pena, 2000), implicating this region in modulation of activation kinetics. Within this stretch of the N terminus lies a “KIKER” sequence (K362‐R366). When the positively charged residues in this sequence were substituted by glutamic acid (EIEEE), a markedly negative (~ −41 mV) voltage‐shift in activation V_0.5_ was observed (Saenen, Labro, Raes, & Snyders, 2006). On the other hand, when the negatively charged glutamate was substituted by arginine (E365R) a marked positive shift in activation V_0.5_ was observed. E365R also showed slightly slowed deactivation (Saenen et al., 2006). The authors of this study concluded that the modulatory effects of the proximal domain on hERG gating are largely electrostatic and localized to the KIKER region (Saenen et al., 2006). The N terminal deletion region of hERG_T_ includes residues 141–350 upstream of the KIKER sequence. It is notable that a larger proximal deletion of residues 141–380 (hERG_TS_), which includes the KIKER sequence, resulted in a marked negative shift of V_0.5_ (−20 mV (Wang & MacKinnon, 2017)). It is plausible, therefore, that the 141–350 deletion influences the availability of KIKER residues to undergo electrostatic interactions with the VSD, producing a modest effect on activation kinetics seen here.

Deletion of the hERG N terminus has been found to alter hERG channel inactivation, likely by removing an N terminus interaction with the internal S4–S5 linker region of the channel (Wang, Trudeau, Zappia, & Robertson, 1998). This is believed to account for altered rectification of heteromeric hERG1a/1b channels, which possess fewer hERG1a N termini to interact with the S4–S5 linker than do tetrameric hERG 1a channels (Sale et al., 2008). We found that inactivation kinetics of the hERG_T_ channel were similar to those of WT hERG, with the only observed difference being a significant acceleration in the recovery of I_hERG_ from inactivation; this may reflect a subtle difference in N terminal interactions with the S4–S5 linker in the hERG_T_ channel.

Slow deactivation of hERG involves an interaction between the N terminal PAS domain and the C terminal cyclic nucleotide binding domain (CNBD; (Gustina & Trudeau, 2011)). The cryo‐EM structures for hERG identified a cytosolic ring structure with the PAS domain of one subunit interacting with the CNBD domain of its neighbor (Wang & MacKinnon, 2017). hERG channels with deletions of CNBD (deletion of residue 749–872) show accelerated deactivation kinetics, whereas deletion of C terminal residues 873–1159 leaves WT activation and deactivation properties unaffected (Gustina & Trudeau, 2011). Muskett *et al* implicated interactions between N terminal residues 1–26 and residues 843, 847 and 850 in the CNBD in I_hERG_ deactivation (Muskett et al., 2011). These residues are proximal to the deleted region from 871 to 1,005 in hERG_T_, however, so it is not obvious how this deletion could influence deactivation of hERG_T_ I_hERG._ On the other hand, residues in the S4–S5 linker provide potential interaction sites for domains that influence stability of the open state and, thereby, deactivation kinetics (Ng et al., 2012). Altered N terminal S4–S5 interactions may therefore account for the slowed I_hERG_ deactivation of hERG_T_ channels. Some studies reporting substantial C terminal deletions in hERG showed these to be associated with reduced current magnitude (Mihic, Chauhan, Gao, Oudit, & Tsushima, 2011; Nof et al., 2010), which is not the case for hERG_T_ here. The basis for this difference is not clear, though it is notable that hERG_T_ involves an excision of part of the C terminus that leaves more than 150 of the final residues intact, while the mutations in these two studies (Mihic et al., 2011; Nof et al., 2010) involved frameshifts and premature stop codons. Thus, they do not mirror the C terminal change made to hERG_T_.

An additional point of note regarding the hERG_T_ construct is that we did not observe significantly reduced functional expression (measured as current density) between WT and hERG_T_ constructs. A number of long QT 2 (LQT2) missense mutations have been reported within the deleted regions (Anderson et al., 2014; Tester, Will, Haglund, & Ackerman, 2005). In the N terminus, this includes G238S, G306W, S320L, R328C (Tester et al., 2005) and P334L (Lupoglazoff et al., 2001). In the distal C terminus this includes A913V and R1005Q (Anderson et al., 2014); the latter residue is part of the RXR C terminal endoplasmic reticulum (ER) retention signal (Kupershmidt et al., 2002). The C terminal deletion in hERG_T_ disrupts this ER retention signal. Clearly the overall effects of deletion of the two stretches of N and C termini in hERG_T_ are not to produce channels with an LQT phenotype.

### 4.2 Implications of hERG_T_ properties for interpretation of pharmacology in light of the hERG cryo‐EM structure

A motivator for this study was an apparent deviation between mutagenesis experiments and docking simulations using the hERG cryo‐EM structure for a minimally structured, high affinity hERG inhibitor (Helliwell et al., 2018). In the cryo‐EM structure, the side chains of F656, which are important for hERG inhibition by multiple drugs (reviewed in Butler et al., 2020), project away from the central pore; a small clockwise rotation of the inner (S6) helix of the hERG pore from its configuration in the cryo‐EM structure was suggested to optimize F656 side chain positions for drug interactions consistent with electrophysiological data (Chen, Seebohm, & Sanguinetti, 2002; Helliwell et al., 2018). An independent study has also reported difficulty using docking to the cryo‐EM structure in recapitulating experimental data that implicate residue T623 (near the base of the selectivity filter/pore helix) in R‐roscovitine binding (Cernuda et al., 2019). Two broad possibilities can account for such differences between in vitro and docking observations: (i) deletions necessary for successful protein purification might affect the channel conformation in ways that are adverse to drug binding and (ii) the channel is essentially normal, but the structure obtained from cryo‐EM may have been captured in a nonoptimal configuration for drug binding (Butler et al., 2020; Robertson & Morais‐Cabral, 2019). In the cryo‐EM structure the S4 voltage sensor was captured in the activated (depolarized), configuration at a nominal potential of 0 mV, and the inner helical gate is open (Wang & MacKinnon, 2017). The information in this study most relevant to this situation is the steady‐state window I_hERG_ (Figure 5). 0 mV is close to the peak of the window current for hERG_T_, while it is a little beyond the peak of the window current for the WT channel. It seems unlikely that this small difference would result in large conformational differences between the pore of WT and hERG_T_ channels. Thus, it seems more likely that the cryo‐EM structure captured a low affinity open state in which the positions of binding residues are nonoptimally configured for interaction with some drugs (Helliwell et al., 2018), although recent studies indicate that the energetic barriers to reorientation of Phe side chains into configurations more optimal for interaction with drugs may be small (Dickson, Velez‐Vega, & Duca, 2020; Negami, Araki, Okuno, & Terada, 2019). There are only limited data available on the pharmacology of hERG_T_ channels (Wang & MacKinnon, 2017) and, with the properties of hERG_T_ I_hERG_ now more fully characterized, we suggest that future comparison be made of effects on drug binding of mutations to key binding residues in the canonical drug binding between WT and hERG_T_. If such mutations similarly affect drug binding to the two channels, this will fully eliminate a role for the deletions in hERG_T_ in influencing drug binding; in that event, the “snapshot” conformation in which the cryo‐EM structure was obtained will be the likely explanation for differences between experimental and docking findings (Cernuda et al., 2019; Helliwell et al., 2018).

### 4.3 Limitations and conclusions

This study has focused on comparing WT and hERG_T_ I_hERG_ under basal conditions. The deleted regions in hERG_T_ contain phosphorylation sites for modulation by protein kinases A and C (PKA and PKC) (Cockerill et al., 2007; Cui, Melman, Palma, Fishman, & McDonald, 2000; Thomas et al., 2003). The well‐known K897T polymorphism, which also introduces a phosphorylation site (Gentile et al., 2008) lies within the portion of the C terminus deleted in hERG_T_. Consequently, while our data indicate that the differences between WT and hERG_T_ I_hERG_ are modest under basal recording conditions, there could be substantial differences in the response of the two channels to PKA or PKC agonism. In this study, we have not compared pharmacological responses of WT and hERG_T_ channels, but this is warranted for future studies. We conclude that at physiological temperature, hERG_T_ channels exhibit rightward voltage‐shifted activation, slowed deactivation and faster recovery from inactivation than does WT hERG. Steady‐state window current is also rightward shifted for hERG_T_ channels. These changes are likely to result, at least in part, from altered electrostatic interactions between the intracellular N terminus and other domains of the hERG channel.

## CONFLICT OF INTEREST

No conflicting interests, financial or otherwise, are declared by the authors.

## AUTHOR CONTRIBUTIONS

Conceptualization and research design: Hancox, Dempsey, and Zhang. Experimentation and data acquisition: Zhang. Data analysis: Zhang. Data interpretation and discussion: Zhang, Hancox, and Dempsey. Wrote or contributed to the writing of the manuscript: Hancox, Zhang, Dempsey.

## ETHICAL STATEMENT

This study was conducted on recombinant hERG channel proteins heterologously expressed in HEK 293 cells and involved no work on human or animal primary tissues.
